# Deaths from Chloroform

**Published:** 1870-07

**Authors:** 


Deaths from Chloroform.—*In Berlin last winter—not reported.*—We learn of it through a private letter from a medical gentleman abroad to a medical friend here.

In the *Chicago Medical Examiner* Dr. Andrews records his belief that, in England and this country, probably not one-fifth of the deaths occurring from anæsthesia are published. The data which we have at hand are not sufficient to warrant any exact approximation of this proportion, but we are sure that very many deaths from chloroform are never made public. Our readers are already familiar with our views on the impropriety of the use of this anæsthetic.—*N. Y. Medical Journal.*

We regret to announce another death from chloroform at University College Hospital on the 3d inst. The morbid appearance found after death are insufficient to explain the fatal result. Chloroform as it happened, was administered in a more diluted form than usual, proportion of the anæsthetic to air being thirty instead of thirty-five minims to each thousand cubic inches of air, as hitherto employed by Mr. Clover, the inventor of the instrument used on the occasion. The verdict given at the inquest, which was merely of a formal character, was "Death by misadventure."—*British Medical Journal, May* 14, 1870.

